# The Emerging Parkinson's Disease Oxylipin‐Ome

**DOI:** 10.1002/advs.202522997

**Published:** 2026-04-22

**Authors:** Julia C. Kelliher, Saranna Fanning

**Affiliations:** ^1^ Ann Romney Center for Neurologic Diseases Department of Neurology Brigham and Women's Hospital and Harvard Medical School Boston Massachusetts USA

**Keywords:** fatty acids, lipids, metabolism, oxylipins, Parkinson's disease

## Abstract

Parkinson Disease (PD) is increasingly considered a proteinopathy and lipidopathy. This proteinopathy+lipidopathy paradigm has been further refined to a fatty acid (FA)‐opathy, centering dysregulated FA metabolism as fundamental in PD lipid dysfunction. FA dysfunction can disrupt alpha‐synuclein (αS)‐membrane interactions, altering αS localization, conformation, and aggregation. Correcting FA dyshomeostasis rescues PD‐associated αS phenotypes and is a promising strategy for disease‐modifying therapeutics. Herein, we consider the impact of PD FA dyshomeostasis in modifying the bioactive oxylipin‐ome. Oxylipin metabolism is complex, and the role of oxylipins in PD is not yet fully determined. This perspective considers PD‐associated differences oxylipin profiles, oxylipin precursor polyunsaturated fatty acids (PUFAs), and oxylipin biosynthetic enzymes in human PD studies to explore a potential PD oxylipin‐ome signature. Founded on disrupted oxylipin and oxylipin precursor PUFA abundance, higher PUFA intake reducing PD risk and progression, and the association between cyclooxygenase (COX) inhibition and lower PD incidence, we posit that the oxylipin‐ome plays a role in PD. Oxylipin metabolism may be a novel target for PD biomarkers and disease‐modifying therapeutics.

## Graphical abstract

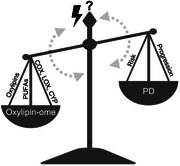

**Abbreviations**
AA: arachidonic acid
AD: alzheimer's disease
Ada: adrenic acid
aLA: alpha‐linolenic acid
ALS: amyotrophic lateral sclerosis
APAU: N‐(1‐acetyl‐4‐piiperidinyl)‐N'tricyclo[3.3.1.1^3,7^]dec‐1‐yl‐urea
BBB: blood‐brain barrier
C: carbon
CI: confidence interval
CNS: central nervous system
COX: cyclooxygenase
CSF: cerebrospinal fluid
CYP: cytochrome p450
DGLA: dihomo‐gamma‐linolenic acid
DHA: docosahexaenoic acid
DiHETE: Dihydroxy‐eicosatetraenoic Acid
DLB: dementia with lewy bodies
ELOVL: elongation of very long‐chain fatty acids
EPA: eicosapentaenoic acid
EpETrE: epoxy‐eicosatrienoic acid
ETA: eicosatetraenoic acid
F: female
FA: fatty acid
FADS: fatty acid desaturase
GBA: glucocerebrosidase
GLA: gamma‐linolenic acid
GPCR: G protein‐coupled receptor
GWAS: genome‐wide association studies
H&Y: Hoehn & Yahr
HDoHE: hydroxy‐docosahexaenoic acid
HEPE: hydroxy‐eicosapetaenoic acid
HETE: hydroxy‐eicosatetraenoic acid
HODE: hydroxy‐octadecadienoic acid
HOTrE: hydroxy‐octadecatrienoic acid
HpETE: hydroperoxyl‐eicosatetraenoic acid
iLBD: incidental lewy body disease
iPSC: induced pluripotent stem cell
KODE: keto‐octadecadienoic acid
LA: linoleic acid
LOX: lipoxygenase
Lt: leukotriene
M: male
MUFA: monounsaturated fatty acid
NC: no change
NSAID: non‐steroidal anti‐inflammatory drug
OR: odds ratio
PD: Parkinson's disease
PG: prostaglandin
PGE2: prostaglandin E2
PLA2: phospholipase A2
PUFA: polyunsaturated fatty acid
RCT: randomized controlled trial
SCD: stearoyl‐CoA‐desaturase
SDA: stearidonic acid
sEH: soluble epoxide hydrolase
THA: tetracosahexaenoic acid
TPA: tetracosapentaenoic acid
TPPU: N‐[1‐(1‐oxopropyl)‐4‐piperidinyl]‐N’‐[4(trifluoromethoxy)phenyl)‐urea
TTA: tetracosatetraenoic acid
Tx: thromboxane
UPDRS: unified Parkinson's disease rating stage
αS: alpha‐synuclein
Ω/ω: omega

## 1 Introduction

Parkinson's Disease (PD) is the second most common and fastest‐growing neurodegenerative disease worldwide [1, 2]. PD incidence varies by gender and race/ethnicity, where age‐adjusted PD rates are higher in men than women and in non‐Hispanic white and Hispanic than Black or Asian individuals [3, 4, 5, 6]. PD is characterized by the loss of dopaminergic neurons in the substantia nigra and the accumulation of intracellular Lewy bodies containing aggregated alpha‐synuclein (αS) [7, 8, 9, 10]. PD has traditionally been considered a proteinopathy with misfolded αS in the hallmark Lewy bodies in the brain. It is now also recognized as a lipidopathy, supported by identification of PD risk loci in lipid metabolism genes through genome‐wide association studies (GWAS), αS‐lipid interactions, αS expression‐induced impacts on the lipidome, co‐localization of lipid membrane fragments with αS in Lewy bodies in the PD brain, bidirectional effects of dysregulated αS and lipid metabolism in PD, lipids recognized as common to several PD pathogenic processes, and amelioration of PD‐associated αS dyshomeostasis by correcting the PD‐associated lipid dyshomeostasis (reviewed in [11]) [10, 11, 12, 13, 14, 15, 16, 17, 18, 19, 20, 21, 22, 23, 24, 25, 26, 27, 28, 29, 30, 31, 32, 33, 34, 35, 36, 37, 38, 39, 40].

**FIGURE 1:**
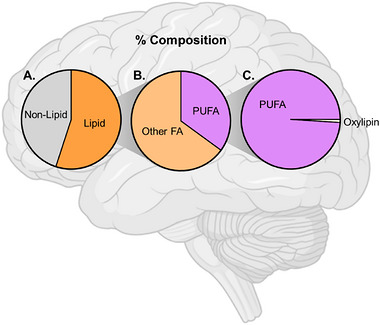
Brain Lipid Composition Schematic. Pie charts represent the relative amount of the dry weight of lipid to non‐lipid (A) [58, 59, 60], polyunsaturated fatty acid (PUFA) to other fatty acid (FA) (B) [67], and an approximation of the proportional abundance of oxylipins to oxylipin precursor PUFAs (C) [68] in the brain. Human brain oxylipins to PUFAs ratio are likely much smaller than what is represented in Figure 1. The brain graphic was obtained from Biorender.

Brain lipids with high structural and functional diversity make up over half of the dry weight of the brain (Figure 1A) and regulate critical physiological brain functions, such as membrane fluidity, neurotransmission, synaptic plasticity, and neuroinflammation [41, 42, 43, 44, 45, 46, 47, 48, 49, 50, 51, 52, 53, 54, 55, 56, 57, 58, 59, 60, 61]. Fatty acid (FA) dyshomeostasis has been identified as an important contributor to the PD‐associated lipid imbalance, refining PD from a lipidopathy to a FA‐opathy [62]. This is supported by studies reporting an altered FA‐ome in PD, newly identified risk loci in FA metabolism genes in PD GWAS, and FA‐induced impacts on αS homeostasis and function (reviewed in [62]). A dysregulated FA‐ome can contribute to PD in a *membrane‐dependent* manner by altering αS‐membrane interactions, which induce changes in αS localization, multimerization, and aggregation [11, 28, 62, 63, 64]. Correcting PD‐associated membrane monounsaturated fatty acid (MUFA) dyshomeostasis corrects membrane FA composition and ameliorates αS pathologies in preclinical mouse and cellular PD models. An inhibitor of the MUFA‐synthesizing enzyme stearoyl‐CoA‐desaturase (SCD) entered PD clinical trials [27, 28, 29, 30, 31, 32, 65, 66]. A disrupted FA‐ome could additionally affect PD pathologies through *membrane‐independent* mechanisms, including through alteration of FA‐derived bioactive oxylipin metabolism.

## 2 Parkinson's Disease: An Oxylipin‐Opathy?

**FIGURE 2:**
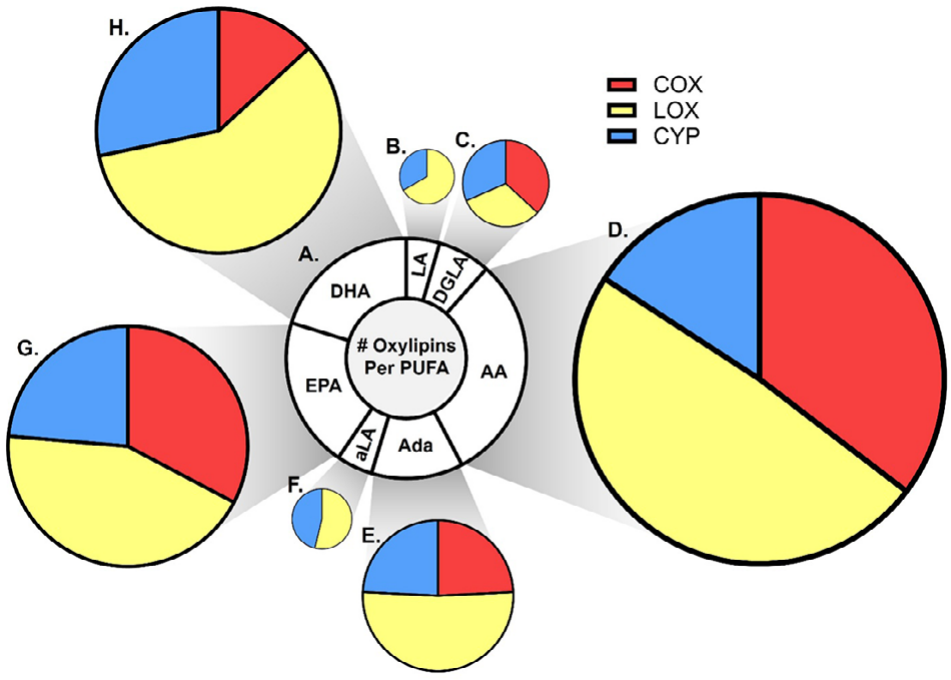
Oxylipin Prevalence by Oxylipin Precursor PUFA and Biosynthesis Pathway. The center pie chart (A) represents the number of known oxylipins produced by each precursor PUFA relative to the total number of unique oxylipins that have been identified to date. In all other pie charts (B–H), the red, yellow, and blue colors indicate the relative proportion of the number of known oxylipins produced from each PUFA via cyclooxygenase (COX), lipoxygenase (LOX), and cytochrome p450 (CYP) enzymatic biosynthetic pathways of production, respectively, relative to the total number of known oxylipins produced by that precursor PUFA. The number of known oxylipins was based on [69]. The size of each (B–H) pie chart corresponds to the relative proportion of the number of oxylipins produced by each precursor PUFA [69]. LA: linoleic acid C18:2ω6. DGLA: dihomo‐gamma‐linolenic acid C20:3ω6. AA: arachidonic acid C20:4ω6. Ada: adrenic acid C22:4ω6. aLA: alpha‐linolenic acid C18:3ω3. EPA: eicosapentaenoic acid C20:5ω3. DHA: docosahexaenoic acid C22:6ω3. ω: omega.

To investigate membrane‐independent mechanisms of FA dyshomeostasis in PD, the dysregulation of polyunsaturated fatty acid (PUFA)‐derived oxylipins is considered herein. Oxylipins are a diverse group of signaling lipids derived from brain‐enriched PUFAs [61, 67] that act as PUFA biological effectors (Figure 1B) (reviewed in [69]). Oxylipins regulate physiological processes that are associated with PD, including inflammation [57, 70, 71, 72, 73, 74, 75, 76, 77], vascular homeostasis [78, 79, 80, 81, 82], pain [72, 83, 84], wound healing [72, 85], and apoptosis [86, 87, 88]. Hundreds of distinct oxylipins are produced from precursor PUFAs, particularly arachidonic acid (AA) C20:4ω6, eicosapentaenoic acid (EPA) C20:5ω3, and docosahexaenoic acid (DHA) C22:6ω3 (Figure 2A) [69, 89]. Oxylipin biosynthesis occurs when cell activation induces phospholipase A2 (PLA2) cleavage of esterified PUFAs from cellular membranes that serve as substrates for cyclooxygenase (COX), lipoxygenase (LOX), and cytochrome p450 (CYP) oxylipin biosynthetic enzymes [69]. Genetic mutations affecting oxylipin production are PD‐associated: mutations in *PLA2G6* that alter PUFA release from cellular membranes are associated with dystonia‐parkinsonism, and polymorphisms in *CYP* genes are associated with sporadic and genetics forms of PD [90, 91, 92, 93, 94]. While oxylipins are low in abundance (Figure 1C), they are highly potent [68, 95, 96, 97]. For example, 10 picograms of DHA C22:6ω3‐derived resolvin D2 inhibits leukocyte infiltration by approximately 70%, and EPA C20:5ω3‐derived 12‐hydroxy‐eicosapentaenoic acid (12‐HEPE) stimulates vasodilation with 10‐fold higher potency than its EPA C20:5ω3 precursor [95, 97]. Oxylipins rapidly recycle between non‐esterified and esterified forms [69, 98]. Non‐esterified oxylipins exert biological effects via intracellular effectors and G protein‐coupled receptors (GPCRs), dysfunction of which is implicated in PD pathogenesis [99, 100, 101, 102]. Esterified oxylipins (>90% of plasma oxylipins) serve as a reservoir pool for the rapid release and sequestration of non‐esterified oxylipins for modulation of oxylipin bioactivity [69, 98, 103, 104]. Lipoproteins, concentrations of which are altered in PD, transport esterified and non‐esterified oxylipins and support propagation of oxylipin signaling cascades across the blood‐brain barrier (BBB), which may be exacerbated by BBB breakdown in neurodegenerative diseases [13, 41, 103, 104, 105, 106].

Oxylipin profiles and the oxylipin inflammatory response are affected by diet/dietary supplements [106, 107, 108, 109, 110, 111, 112, 113, 114, 115, 116], sex [105, 117, 118], aging [108, 118], and disease [110, 111, 119, 120, 121]. Most oxylipin disease research thus far has focused on oxylipins in cardiovascular [122], metabolic diseases [123], and cancer [124]. Recent studies have implicated altered oxylipin metabolism in neurodegenerative diseases, including PD [104, 125, 126, 127, 128], Alzheimer's Disease (AD) [120, 121], and amyotrophic lateral sclerosis (ALS) [129]. Though understanding of the role of oxylipins in PD is still emerging, we propose that oxylipin metabolism in the PUFA‐rich brain contributes to PD supported by differences in (1) oxylipin‐omic profiles, (2) oxylipin precursor PUFAs, and (3) oxylipin biosynthetic enzymes in PD.

### 2.1 The Oxylipin‐Ome is Disrupted in PD

**FIGURE 3:**
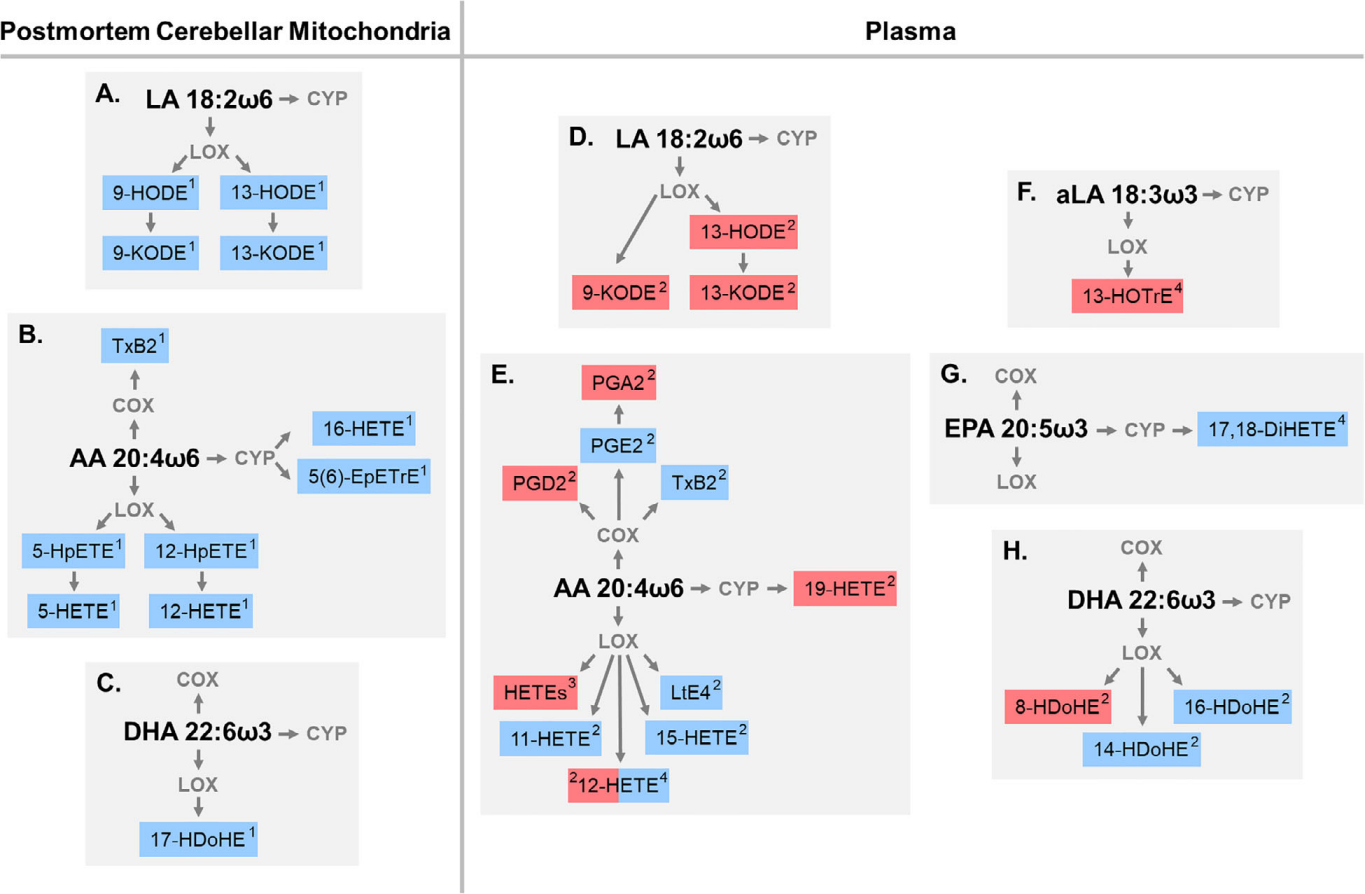
Oxylipin Profile Differences in Sporadic Parkinson's Disease. Red and blue colors indicate significantly higher or lower oxylipin concentrations, respectively, in biological samples of individuals with PD compared to non‐PD individuals. Oxylipins were quantified from postmortem cerebellar mitochondria of female subjects (A–C) or from plasma of both male and female subjects (D–H). Superscript numbers indicate the publication that reported the finding: ^1^: [125]. ^2^: [128]. ^3^: [126]. ^4^: [127]. LA: linoleic acid. AA: arachidonic acid. aLA: alpha‐linolenic acid. EPA: eicosapentaenoic acid. DHA: docosahexaenoic acid. COX: cyclooxygenase. LOX: lipoxygenase. CYP: cytochrome p450. HODE: hydroxy‐octadecadienoic acid. KODE: keto‐octadecadienoic acid. Tx: thromboxane. HpETE: hydroperoxyl‐eicosatetraenoic acid. HETE: hydroxy‐eicosatetraenoic acid. PG: prostaglandin. Lt: leukotriene. HOTrE: hydroxy‐octadecatrienoic acid. DiHETE: Dihydroxy‐eicosatetraenoic acid. HDoHE: hydroxy‐docosahexaenoic acid.

Oxylipin profiling studies of individuals with and without PD is a direct way to identify PD‐associated differences in the oxylipin‐ome. However, few studies have reported on oxylipin profiles in PD biological samples, and no study to date has described the PD oxylipin‐ome in genetic forms of PD. The sole oxylipin‐omic study of PD postmortem brain measured oxylipins in cerebellar mitochondria, identifying lower concentrations of LA C18:2ω6‐ and AA C20:4ω6‐derived oxylipins in mitochondria from females with PD compared to females without PD (Figure 3A–C) [125]. The concentration of only one oxylipin, AA C20:4ω6/COX‐derived prostaglandin E2 (PGE2), has been reported in the cerebrospinal fluid (CSF) of PD subjects, where total PGE2 concentrations did not differ between subjects with or without PD [130]. In plasma, individuals with PD had higher LA C18:2ω6/LOX‐derived oxylipin concentrations, altered AA C20:4ω6‐derived oxylipins produced via all enzymatic pathways, higher aLA C18:3ω3/LOX‐derived 13‐hydroxy‐octadecatrienoic acid (13‐HOTrE), and predominantly lower EPA C20:5ω3‐ and DHA C22:6ω3‐derived oxylipins (Figure 3D–H) [126, 127, 128]. Concentrations of AA C20:4ω6‐derived oxylipins made via all COX, LOX, and CYP enzymatic biosynthetic pathways were altered in PD postmortem brain mitochondria and plasma, with AA C20:4ω6‐derived oxylipins lower in cerebellar mitochondria and identified as both higher and lower in the plasma of PD vs non‐PD subjects (Figure 3B,E) [125, 126, 127, 128]. Plasma aLA C18:3ω3‐derived 13‐HOTrE concentrations were proposed (100% sensitivity, 82.4% specificity) as a biomarker for identifying PD [127]. To the best of our knowledge, no study has yet reported esterified oxylipin concentrations in any biological sample from subjects with or without PD.

### 2.2 Oxylipin Precursor PUFA Abundance is Altered in PD

Oxylipin precursor PUFA availability impacts the oxylipin‐ome and associated biological effects. Details on the complexity of PUFA metabolism are beyond the scope of this perspective (see [41, 131], among others for reviews). Briefly, PUFAs are classified into omega (ω)6 and ω3 groups based on the position of their terminal double bond. Ω6 and ω3 PUFAs are broadly categorized as pro‐ or anti‐inflammatory, respectively, depending on how they are metabolized [41, 131]. PUFAs are highly enriched in the brain, making up over a third of all brain FAs (Figure 1B) [67]. The predominant brain PUFAs are the ω6 PUFA arachidonic acid (AA) C20:4ω6 and the ω3 PUFA DHA C22:6ω3, which, along with EPA C20:5ω3, produce the majority of known oxylipins (Figure 2) [67, 69]. AA C20:4ω6 and DHA C22:6ω3 maintain essential physiological roles in brain development, learning and memory, neurotransmission, dopamine metabolism, and neuroinflammation among other brain functions [41, 132, 133, 134, 135, 136, 137, 138, 139, 140, 141, 142, 143, 144, 145]. Neurons are dependent on a continuous supply of AA C20:4ω6 and DHA C22:6ω3 predominantly from the periphery through dietary intake or hepatic conversion of dietary essential PUFAs linoleic acid (LA) C18:2ω6 and alpha‐linolenic acid (aLA) C18:3ω3 via fatty acid desaturase (FADS) and elongation of very long‐chain fatty acids (ELOVL) enzymes, or, to a lesser extent, from PUFA desaturation and elongation in glial cells [41, 146, 147]. Herein, we reviewed PD‐associated differences in PUFA concentrations in the central nervous system (CNS) and periphery of individuals with and without PD. PUFA profiling studies identified altered PUFA concentrations in PD postmortem brain tissue, cerebrospinal fluid (CSF), and plasma relative to those without PD (discussed in Sections 2.2.1–2.2.3) [33, 125, 126, 127, 128, 148, 149, 150, 151, 152, 153, 154, 155, 156, 157, 158, 159]. To the best of our knowledge, there is no available information regarding potential PD‐associated concentration alterations in any PD biological sample type for several PUFAs, including gamma‐linolenic acid (GLA) C18:3ω6, tetracosatetraenoic acid (TTA) C24:4ω6, tetracosapentaenoic acid (TPA) C24:5ω6, stearidonic acid (SDA) C18:4ω3, eicosatetraenoic acid (ETA) C20:4ω3, TPA C24:5ω3, and tetracosahexaenoic acid (THA) C24:6ω3.

#### 2.2.1 PD‐Associated Differences in Brain PUFA Concentrations are Region and PD Type‐Specific

Brain PUFA concentrations directly impact brain oxylipin metabolism and differ in PD postmortem brain tissue (Tables 1 and 2) [33, 125, 148, 149, 150, 151, 154]. PD is associated with lower concentrations of essential PUFAs LA C18:2ω6 and aLA C18:3ω3, particularly in the substantia nigra (Tables 1 and 2) [149, 150, 151]. The PD substantia nigra has lower concentrations of ω6 and ω3 PUFAs at the beginning and end of PUFA metabolism pathways and higher Dihomo‐gammalinoleic acid DGLA C20:3ω6 and Docosapentaenoic acid DPA C22:5ω3 concentrations than in the non‐PD substantia nigra (Tables 1 and 2) [149, 150]. Sporadic PD is predominantly associated with altered brain concentrations of ω6 PUFAs, with brain PUFAs being higher or lower depending on the PUFA and brain region (Table 1) [33, 148, 150, 151, 154]. Individuals with incidental Lewy body disease (iLBD) and dementia with Lewy bodies (DLB) have differences in brain ω6 and ω3 PUFAs, where all quantified brain ω6 PUFAs except DGLA C20:3ω6 are lower in subjects with iLBD/DLB compared to non‐PD subjects (Table 2) [149, 151]. Some C20 and C22 PUFA concentrations are higher in the PD brain, depending on brain region and PD type (Tables 1 and 2). Differences in PD‐associated brain PUFAs may be due to small study sample sizes, different brain regions profiled, and postmortem interval artefacts.

**TABLE 1:** Sporadic Parkinson's Disease‐Associated Concentration Differences in Postmortem Brain Polyunsaturated Fatty Acid (PUFAs). Significantly higher (red) and lower (blue) PUFA concentrations in postmortem brain of subjects with sporadic PD compared to those without PD are listed in Table 1. Ω: Omega. LA: Linoleic acid. DGLA: Dihomo‐gamma‐linolenic acid. AA: Arachidonic acid. Ada: Adrenic acid. DPA: Docosapentaenoic acid. DHA: Docosahexaenoic acid. M: Male. F: Female.

Reference	Subjects	Ω	Chain length	Double bonds	PUFA	Brain region	Sporadic PD vs. Non‐PD
[150]	Non‐PD n = 19 (8M/11F); PD n = 18 (9M/9F)	6	18	2	LA	Substantia nigra	Lower
[151]	Non‐PD n = 11 (5M/6F); PD n = 8 (5M/3F)					Frontal cortex lipid rafts	Lower
[148]	Non‐PD n = 10 (5 M/5F); PD n = 9 (7M/2F)		20	3	DGLA	Anterior cingulate cortex	Lower
[151]	Non‐PD n = 11 (5M/6F); PD n = 8 (5M/3F)					Frontal cortex lipid rafts	Lower
[154]	Non‐PD n = 9; PD n = 12		20	4	AA	Temporal cortex	Higher
[125]	Non‐PD n = 15 (10M/5F); Braak 5–6 PD n = 10 (5M/5F)					Cerebellar mitochondria	Lower
[125]	Non‐PD n = 10M; Braak 3‐4 PD n = 5M					Cerebellar mitochondria	Higher
[151]	Non‐PD n = 11 (5M/6F); PD n = 8 (5M/3F)					Frontal cortex lipid rafts	Lower
[33]	Non‐PD n = 7; PD n = 7		22	4	Ada	Brain	Higher
[148]	Non‐PD n = 10 (5M/5F); PD n = 9 (7M/2F)					Anterior cingulate cortex	Lower
[148]	Non‐PD n = 10 (5M/5F); PD n = 9 (7M/2F)			5	DPA	Anterior cingulate cortex	Higher
[151]	Non‐PD n = 11 (5M/6F); PD n = 8 (5M/3F)					Frontal cortex lipid rafts	Lower
[33]	Non‐PD n = 7; PD n = 7	3	22	6	DHA	Brain	Higher
[151]	Non‐PD n = 11 (5M/6F); PD n = 8 (5M/3F)					Frontal cortex lipid rafts	Lower

**TABLE 2:** Incidental Lewy Body Disease (iLBD)‐ and Dementia with Lewy Bodies (DLB)‐Associated Concentration Differences in Postmortem Brain Polyunsaturated Fatty Acid (PUFAs). Significantly higher (red) and lower (blue) PUFA concentrations in the postmortem brain of subjects with iLBD/DLB compared with those without are listed in Table 2. Ω: Omega. LA: Linoleic acid. DGLA: Dihomo‐gamma‐linolenic acid. AA: Arachidonic acid. Ada: Adrenic acid. DPA: Docosapentaenoic acid. aLA: Alpha‐linolenic acid. DHA: Docosahexaenoic acid. M: Male. F: Female.

Reference	Subjects	Ω	Chain length	Double bonds	PUFA	Brain region	Incidental PD/DLB vs. Non‐PD
[151]	Non‐PD n = 11 (5M/6F); iLBD n = 8 (6M/2F)	6	18	2	LA	Frontal cortex lipid rafts	Lower
[149]	Non‐PD n = 3 (2M/1F); iLBD n = 4 (3M/1F); DLB n = 4 (2M/2F)		20	3	DGLA	Substantia nigra	Higher
[151]	Non‐PD n = 11 (5M/6F); iLBD n = 8 (6M/2F)			4	AA	Frontal cortex lipid rafts	Lower
[149]	Non‐PD n = 3 (2M/1F); iLBD n = 4 (3M/1F); DLB n = 4 (2M/2F)		22	4	Ada	Cerebral cortex	Lower
[149]	Non‐PD n = 3 (2M/1F); iLBD n = 4 (3M/1F); DLB n = 4 (2M/2F)			5	DPA	Substantia nigra	Lower
[149]	Non‐PD n = 3 (2M/1F); iLBD n = 4 (3m/1F); DLB n = 4 (2M/2F)					Amygdala	Lower
[151]	Non‐PD n = 11 (5M/6F); iLBD n = 8 (6M/2F)					Frontal cortex lipid rafts	Lower
[149]	Non‐PD n = 3 (2M/1F); iLBD n = 4 (3M/1F); DLB n = 4 (2M/2F)	3	18	3	aLA	Substantia nigra	Lower
[149]	Non‐PD n = 3 (2M/1F); iLBD n = 4 (3M/1F); DLB n = 4 (2M/2F)		22	5	DPA	Substantia nigra	Higher
[149]	Non‐PD n = 3 (2M/1F); iLBD n = 4 (3M/1F); DLB n = 4 (2M/2F)					Amygdala	Higher
[151]	Non‐PD n = 11 (5M/6F); iLBD n = 8 (6M/2F)					Frontal cortex lipid rafts	Lower
[149]	Non‐PD n = 3 (2 M/1F); iLBD n = 4 (3M/1F); DLB n = 4 (2M/2F/)			6	DHA	Substantia nigra	Lower
[149]	Non‐PD n = 3 (2 M/1F); iLBD n = 4 (3M/1F); DLB n = 4 (2F/2 M)					Cerebral cortex	Higher
[149]	Non‐PD n = 3 (2M/1F); iLBD n = 4 (3M/1F); DLB n = 4 (2M/2F)					Amygdala	Higher
[151]	Non‐PD n = 11 (5M/6F); iLBD n = 8 (6M/2F)					Frontal cortex lipid rafts	Lower

#### 2.2.2 PD‐Associated Differences in **CSF** PUFA Profiles Depend on PD Type

Limited information is available on differences in CSF PUFA composition in PD due to the invasive nature of CSF collection and the low abundance of CSF PUFAs. Sporadic PD has been associated with higher CSF concentrations of ω6 PUFAs with 4 double bonds (Table 3) [152, 158]. CSF ω6 and ω3 PUFA concentrations may be lower in glucocerebrosidase (GBA)‐PD (Table 3) [155]. More studies are needed to determine CSF PUFA concentrations in PD.

**TABLE 3:** Parkinson's Disease‐Associated Concentration Differences in Cerebrospinal Fluid (CSF) Polyunsaturated Fatty Acids (PUFAs). Significantly higher (red) or lower (blue) PUFA concentrations in CSF of subjects PD compared to those without are listed in Table 3. Ω: Omega. GBA: Glucocerebrosidase. LA: Linoleic acid. AA: Arachidonic acid. Ada: Adrenic acid. EPA: Eicosapentaenoic acid. DHA: Docosahexaenoic acid. M: Male. F: Female.

Reference	Subjects	pd type	Ω	Chain length	Double bonds	PUFA	PD vs. Non‐PD
[158]	Non‐PD n = 95 (36M/59F); PD n = 31 (22M/9F)	Sporadic	6	20	4	AA	Higher
[152]	Postmortem non‐PD n = 10 (6M/4F); PD n = 20 (13 M/7F)			22	4	Ada	Higher
[155]	Non‐PD n = 30 (11M/19F); PD n = 8 (4M/4F; L444P mutation n = 5; N370S mutation n = 3)	GBA‐PD	6	18	2	LA	Lower
				20	4	AA	Lower
			3	20	5	EPA	Lower
				22	6	DHA	Lower

#### 2.2.3 Plasma PUFA Concentrations are Lower in Sporadic PD

PUFA profiling of plasma is more feasible than CSF due to the relatively non‐invasive nature of acquiring plasma. However, differences in plasma PUFA concentrations may not represent PUFA profile changes in the CNS. For example, contrary to higher ω6 PUFA concentrations in PD CSF, plasma ω6 and ω3 PUFA concentrations have been reported to be predominantly lower in sporadic PD vs non‐PD subjects (Tables 3 and 4) [126, 127, 128, 152, 157, 158, 159, 160]. Essential PUFAs LA C18:2ω6 and aLA C18:3ω3 are lower in sporadic PD plasma [157, 160]. LA C18:2ω6 and aLA C18:3ω3 metabolism pathways have been implicated in PD‐associated disruptions in plasma metabolites [153]. Most studies identified lower plasma AA C20:4ω6 concentrations in PD, though one study reported higher plasma AA C20:4ω6 in subjects with PD compared to non‐PD and that plasma AA C20:4ω6 concentrations were highly effective (100% sensitivity, 94.1% specificity) in identifying PD cases (Table 4) [126, 127, 128, 159, 160]. Plasma EPA C20:5ω3 concentrations are altered in PD (Table 4) [128, 153]. It is possible that lower plasma PUFA concentrations in sporadic PD, particularly of AA C20:4ω6 and Ada C22:4ω6, could be due to PUFA sequestration in the CNS.

**TABLE 4:** Sporadic Parkinson's Disease‐Associated Concentration Differences in Plasma Polyunsaturated Fatty Acids (PUFAs). Significantly higher (red) and lower (blue) PUFA concentrations in plasma of subjects with sporadic PD compared to those without PD are listed in Table 4. Ω: Omega. LA: Linoleic acid. AA: Arachidonic acid. EPA: Eicosapentaenoic acid. DHA: Docosahexaenoic acid. M: Male. F: Female.

Reference	Sporadic PD subjects	Ω	Chain length	Double bonds	PUFA	PD vs. Non‐PD
[157]	Non‐PD n = 20 (10M/10F); PD n = 20 (10M/10F)	6	18	2	LA	Lower
[160]	Non‐PD n = 33 (10M/23F); PD n = 38 (17M/21F)					Lower
[128]	Non‐PD n = 36 (12M/24F); PD n = 73 (41M/32F)		20	4	AA	Lower
[126]	Non‐PD n = 61 (30M/31F); PD n = 61 (28M/33F)					Lower
[159]	Non‐PD n = 169 (94M/75F); PD n = 223 (124M/99F)					Lower
[160]	Non‐PD n = 33 (10 M/23F); PD n = 38 (17 M/21F)					Lower
[127]	Non‐PD n = 54 (27M/27F); PD n = 42 (28M/14F)					Higher
[160]	Non‐PD n = 33 (10M/23F); PD n = 38 (17M/21F)	3	18	3	aLA	Lower
[128]	Non‐PD n = 36 (12M/24F); PD n = 73 (41M/32F)		20	5	EPA	Higher
[153]	Non‐PD n = 12 (7M/5F); PD n = 25 (17M/8F)					Higher
[159]	Non‐PD n = 169 (94M/75F); PD n = 223 (124M/99F)					Lower
[127]	Non‐PD n = 54 (27M/27F); PD n = 42 (28M/14F)		22	6	DHA	Lower
[159]	Non‐PD n = 169 (94M/75F); PD n = 223 (124M/99F)					Lower

### 2.3 Oxylipin Precursor PUFA Intake May Impact PD Risk and Progression

Plasma PUFA concentrations are predominantly lower in individuals with sporadic PD than those without PD, and lower plasma concentrations of PUFAs and ω6 PUFAs are associated with a higher risk of PD [125, 126, 127, 128, 150, 151, 155, 157, 159, 160, 161]. PUFA concentrations in humans are highly affected by diet because (1) mammals lack the necessary enzymes to synthesize essential PUFAs LA C18:2ω6 and aLA C18:3ω3 and (2) PUFA metabolism involves competition between ω6 and ω3 PUFAs for the same FADS and ELOVL enzymes [162]. A dietary intake ratio of approximately 1:1 ω6:ω3 PUFAs has been associated with reduced risk of chronic diseases and better health outcomes, whereas diets with higher ω6:ω3 PUFA ratios are associated with increased neurodegenerative disease risk [162, 163, 164, 165]. Notably, the Western diet has an approximate ratio of about 20:1 ω6:ω3 PUFAs [162, 165, 166].

#### 2.3.1 Oxylipin Precursor PUFA Consumption May Reduce PD Risk

**FIGURE 4:**
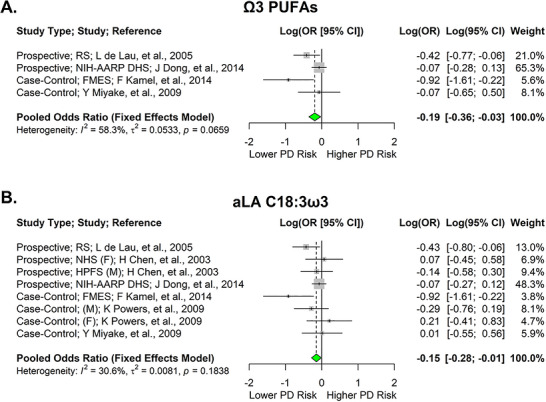
Meta‐Analyses suggest Higher Omega (Ω)3 Polyunsaturated Fatty Acid (PUFA) Intake is Associated with Reduced Parkinson's Disease (PD) Risk. Forest plots indicate the log odds ratios (OR),95% confidence intervals (CI), and study weights of each report analyzed in the meta‐analyses examining how PUFA intake affects PD risk. Horizontal lines in the plots correspond to the 95% CI and the square on each line represents the study weight. The green diamond at the bottom of each plot indicates a significantly lower pooled OR with 95% CI as calculated using a fixed effects model for assessing how omega (ω)3 PUFA (A) and alpha‐linolenic acid (aLA) C18:3ω3 (B) intake affects PD risk.

Numerous prospective and case‐control studies have examined how PD risk is affected by dietary PUFA intake [167, 168, 169, 170, 171, 172, 173, 174, 175, 176]. Higher adherence to the PUFA‐rich Mediterranean diet is associated with reduced PD risk [167, 168]. Reported results on the effects of PUFA consumption on PD risk are varied, which may partially be due to the difficulty of precisely quantifying intake of individual PUFAs using food surveys, some of which require subjects to recall foods consumed years or decades earlier. To overcome this limitation, we assessed the pooled effect of PUFA consumption on PD risk by conducting meta‐analyses on studies that examined the relationship between PUFA intake and PD incidence [169, 170, 171, 172, 173, 174, 175, 176]. Because PUFAs maintain diverse biological functions, such as opposing effects on inflammation, we sought to assess how consumption of different types of PUFAs may differentially affect PD risk. We performed meta‐analyses on studies reporting how PD risk is affected by dietary intake of grouped PUFAs (total PUFAs, ω6 PUFAs, ω3 PUFAs) and individual PUFAs (LA C18:2ω6, AA C20:4ω6, aLA C18:3ω3, EPA C20:5ω3, DPA C22:5ω3, DHA C22:6ω3). Meta‐analyses were conducted using “meta” and “metafor” packages in R (version 4.5.1) on reported log‐transformed odds ratios (OR) and the standard error of reported log‐transformed 95% confidence intervals (CI). Pooled OR in meta‐analyses were calculated using fixed effects models. Study weight in pooled OR calculations in meta‐analyses was determined using the calculated standard error of the reported log‐transformed 95% CI for each study. Due to well‐established sex differences in PUFA metabolism, we included sex‐specific reported OR in meta‐analyses whenever possible to assess potentially sexually dimorphic associations between PUFA intake and PD risk [177, 178, 179, 180, 181, 182]. Meta‐analyses suggest that higher consumption of ω3 PUFAs and essential PUFA aLA C18:3ω3 is associated with lower PD risk (Figure 4A–D) [169, 170, 172, 173, 174, 175]. Higher aLA C18:3ω3 consumption may reduce PD risk in men more than women (Figure 4D) [170, 174]. While meta‐analyses did not identify any other grouped or individual PUFA as significantly impacting PD risk, individual studies have reported significant effects of total PUFA, ω6 PUFA, LA C18:2ω6, and AA C20:4ω6 consumption on PD risk (Figures S1 and S2). Significant heterogeneity in pooled OR for meta‐analyses in supplementary figures may be due in part to study differences in design (e.g. study type, follow‐up intervals between assessment of dietary intake and PD incidence, type of food frequency questionnaire utilized), population demographics (e.g. sex, age, race/ethnicity), population lifestyle behaviors (e.g. cigarette smoking, alcohol consumption, coffee intake), and methods of PD case ascertainment (e.g., treating neurologist‐confirmed, self‐reported). Funnel plots for each meta‐analysis assessing the effects of dietary PUFA consumption on PD risk are shown in Figure S3. It is unknown how dietary intake of other PUFAs, e.g., GLA C18:3ω6, DGLA C20:3ω6, TTA C24:4ω6, TPA C24:5ω6, DPA C22:5ω6, SDA C18:4ω3, ETA C20:4ω3, TPA C24:5ω3, or THA C24:6ω3 affect PD risk.

#### 2.3.2 Oxylipin Precursor PUFA Treatment Prevents PD Progression in PD Clinical Trials

PUFA supplementation corrects PD readouts, including dopaminergic neuronal death and motor deficits, in animal models of PD [183, 184, 185, 186, 187, 188, 189, 190, 191, 192, 193, 194, 195]. PD randomized controlled trials (RCT) examined whether PUFA supplementation could be an effective treatment for PD (Table 5) [196, 197, 198, 199]. Three of four RCT reported that PUFA supplementation prevented PD progression as assessed by Unified Parkinson's Disease Rating Stage (UPDRS) scores [196, 197, 198, 199]. PUFA supplementation was associated with reduced inflammation and depressive symptoms and increased antioxidant capacity in PD [196, 199, 200]. The sole PD clinical trial that did not observe a significant effect of PUFA supplementation on PD progression used LA C18:2ω6‐rich corn oil as their placebo [197]. In this study, neither the treatment nor the placebo groups exhibited significant PD progression, perhaps supporting a beneficial effect of PUFA supplementation in both treatment and placebo groups [197]. It is unknown how PUFA supplementation affected oxylipin metabolism in these RCT subjects. Understanding how modulating oxylipin metabolism by PUFA supplementation in PD may contribute to new therapeutics to reduce disease progression.

**TABLE 5:** Randomized Controlled Trials (RCT) of Polyunsaturated Fatty Acid (PUFA) Supplementation on Parkinson's Disease Progression. Characterization of PD clinical trials assessing the therapeutic potential of PUFA supplementation on Unified Parkinson's Disease Rating Scale (UPDRS) or Hoehn &Yahr scale (H&Y). ω: Omega. LA: Linoleic acid. DGLA: Dihomo‐gamma‐linolenic acid. GLA: Gamma‐linolenic acid. aLA: Alpha‐linolenic acid. EPA: Eicosapentaenoic acid. DHA: Docosahexaenoic acid. M: Male. F: Female. NC: No change.

Reference	RCT location	PD subjects	Placebo	Treatment	Treatment duration	PD variable	Treatment outcome
[197]	Italy	Placebo n = 12 (6M/6F); Treatment n = 12 (7/5F)	Equicaloric corn oil (high in LA C18:2ω6)	290 mg/d EPA C20:5ω3 + 800 mg/d DHA C22:6ω3	6 months	UPDRS	NC
	Italy	Placebo n = 12 (6M/6F); Treatment n = 12 (7M/5F)	Equicaloric corn oil (high in LA C18:2ω6)	290 mg/d EPA C20:5ω3 + 800 mg/d DHA C22:6ω3	6 months	H&Y	NC
[196]	Iran	Placebo n = 30 (21M/9F); Treatment n = 30 (21M/9F)	Not specified	1000 mg flaxseed oil ω3 PUFA (high in aLA C18:3ω3) + 400 IU vitamin E	3 months	UPDRS	Lower
[199]	Iran	Placebo n = 20; Treatment n = 20	Paraffin	1000 mg flaxseed oil ω3 PUFA (high in aLA C18:3ω3) + 400 IU vitamin E	3 months	UPDRS	Lower
[198]	Cyprus	Placebo n = 20 (12M/8F); Treatment n = 20 (9M/11F)	Virgin olive oil	3150 mg LA C18:2ω6 + 1800 mg GLA C18:3ω6 + 810 mg EPA C20:5ω3 + 4140 mg DHA C22:6ω3 + antioxidants (0.6 mg vitamin A, 22 mg vitamin E, 760 mg γ‐tocopherol)	30 months	UPDRS	Lower

### 2.4 Oxylipin Biosynthetic Pathways in PD

Oxylipin precursor PUFA availability determines which oxylipins are produced and the resulting biological implications of activated oxylipin signaling cascades [106, 107, 110, 112, 201]. Oxylipin generation also depends on the pathway through which PUFAs are metabolized via oxylipin biosynthetic COX, LOX, and CYP enzymes [69]. Oxylipins produced from the same precursor PUFA can exert opposing biological effects [70, 78, 202, 203, 204, 205]. For example, AA C20:4ω6 metabolism via COX/LOX pathways produces oxylipins with potent pro‐inflammatory and vasoconstrictive effects, whereas AA C20:4ω6 processing through the CYP pathway generates oxylipins with highly anti‐inflammatory and vasoconstrictive effects [70, 78, 202, 203, 204, 205]. In general, the COX pathway of oxylipin production generates pro‐inflammatory oxylipins (e.g., prostaglandins), the LOX pathway produces pro‐ and anti‐inflammatory oxylipins (e.g., leukotrienes and hydroxy‐docosahexaenoic acids [HDoHEs]), and the CYP pathway synthesizes anti‐inflammatory oxylipins (e.g., epoxy‐eicosatrienoic acids [EpETrEs]) [203, 205, 206, 207]. The impact of oxylipin biosynthetic enzymatic pathways in PD can be indirectly assessed through examination of the effects of oxylipin biosynthetic enzyme inhibitors on PD risk and whether individuals with or without PD have differences in oxylipin biosynthetic enzyme expression.

#### 2.4.1 Inhibiting the Oxylipin Biosynthetic COX Pathway Reduces PD Risk

**FIGURE 5:**
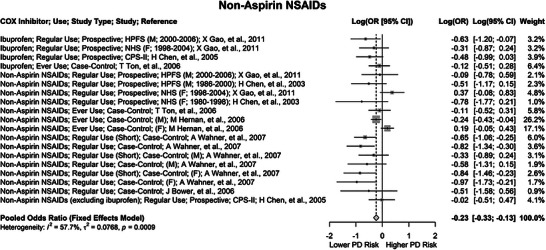
Cyclooxygenase (COX) Inhibitor is Associated with Lower Parkinson's Disease (PD) Risk. Forest plots indicate the log odds ratios (OR),95% confidence intervals (CI), and study weights of reports analyzed in the meta‐analysis to examine the impact of COX inhibitor use on PD risk. Horizontal lines in the plots correspond to the 95% CI and the square on each line represents the weight of that study on the overall pooled OR. The green diamond at the bottom of each plot shows a significantly lower pooled OR with 95% CI as calculated using a fixed effects model for assessing how non‐aspirin non‐steroidal anti‐inflammatory drug (NSAID) use affects PD risk.

Most research on oxylipin biosynthetic enzymatic pathways in PD has focused on the COX pathway due to the well‐characterized pro‐inflammatory effects of AA C20:4ω6 COX‐derived prostaglandins [208, 209, 210, 211, 212, 213, 214, 215, 216, 217, 218]. COX inhibitor treatment reduces AA C20:4ω6‐induced production of PGE2 in postmortem PD substantia nigra [219]. We conducted meta‐analyses of studies examining associations between COX inhibitor use and PD incidence to investigate the pooled effect of COX inhibition on PD risk using the same methodology as previously described for meta‐analyses assessing associations between PUFA intake and PD risk (Figure 5; Figure S4) [208, 209, 210, 211, 212, 214, 215, 216, 217, 218, 220]. Due to known sex differences in COX‐derived oxylipin signaling and sex‐dependent effects of COX inhibition, we included sex‐specific reported OR in meta‐analyses whenever possible to determine potential sex‐dependent associations between COX inhibitor use and PD risk [221, 222, 223, 224]. Meta‐analyses identified a significant association between higher COX inhibitor use of non‐aspirin non‐steroidal anti‐inflammatory drugs (NSAIDs) and lower PD risk, which may be more pronounced in men (Figure 5; Figure S4) [208, 209, 210, 211, 212]. Similarly to meta‐analyses on dietary PUFA intake and PD risk, significant heterogeneity in pooled OR for meta‐analyses on COX inhibitor use and PD risk may be due in part to study differences as previously outlined. Funnel plots for each meta‐analysis assessing the effects of COX inhibitor use on PD risk are shown in Figure S5. COX expression has been identified as altered in the PD brain [125, 225]. In PD postmortem substantia nigra, COX‐1 and COX‐2 expression is higher in microglia but not neurons in the non‐PD substantia nigra [225]. COX‐2 expression is lower in postmortem cerebellar mitochondria of Braak stage 5‐6 males with PD relative to non‐PD males [125]. COX‐2 expression in postmortem cerebellar cortex did not differ between subjects with or without PD [156]. COX‐2 RNA expression is higher in fibroblasts from subjects with sporadic and genetic forms of PD than non‐PD subjects [226].

#### 2.4.2 LOX and CYP Oxylipin Biosynthetic Pathways Remain to be Explored in PD

Little is known about PD‐associated differences in the pro‐ and anti‐inflammatory LOX and anti‐inflammatory CYP oxylipin biosynthetic pathways. However, greater intake of caffeine, which contains high concentrations of the 5‐LOX inhibitor caffeic acid, reduces PD risk [227, 228, 229]. 5‐LOX expression in postmortem cerebellar cortex does not differ between subjects with or without PD [156]. Expression of soluble epoxide hydrolase (sEH), which dampens anti‐inflammatory effects of CYP‐derived epoxide oxylipins by converting them to more biologically inert diol oxylipins, is higher in DLB than non‐PD subjects and is higher in induced pluripotent stem cell (iPSC)‐derived neurons from an individual with a genetic form of PD than non‐PD neurons [230]. Although preclinical studies are outside the scope of this perspective, it is important to note that LOX inhibitors, such as 1‐[(4‐chlorophenyl)methyl]‐3‐[(1,1‐dimethylethyl)thio]‐α,α‐dimethyl‐5‐(1‐methylethyl)‐1H‐indole‐2‐propanoic acid MK‐886, and sEH inhibitors, including N‐[1‐(1‐oxopropyl)‐4‐piperidinyl]‐N’‐[4(trifluoromethoxy)phenyl)‐urea (TPPU) or N‐(1‐acetyl‐4‐piperidinyl)‐N’‐tricyclo[3.3.1.1^3,7^]dec‐1‐yl‐urea (APAU), improve PD phenotypes like survival and neurotoxicity in animal and cellular PD models, and LOX inhibitors are being developed for PD clinical trials [230, 231, 232, 233, 234, 235, 236].

## 3 Discussion

Founded on studies reviewed herein, the oxylipin‐ome may be different in individuals with PD, as evidenced by PD‐associated differences in (1) oxylipin concentrations, (2) oxylipin precursor PUFA abundance, and (3) oxylipin biosynthetic enzyme expression. Modulators of oxylipin metabolism may be useful for reducing PD incidence and stabilizing PD progression. Studies of sporadic PD have identified disrupted CNS metabolism of ω6 PUFAs and peripheral metabolism of ω6 and ω3 PUFAs in PD. So far, AA C20:4ω6 is the only PUFA reported to have altered abundance in sporadic PD brain, CSF, and plasma, where concentrations of AA C20:4ω6 appear to be higher in the CNS and lower in the periphery of individuals with sporadic PD. Though far less studied, iLBD, DLB, and GBA‐PD appear to be associated with altered CNS metabolism of ω6 and ω3 PUFAs. Essential PUFAs LA C18:2ω6 and aLA C18:3ω3 are lower in PD brain, CSF, and plasma. The essential PUFA aLA C18:3ω3 has been reported as lower in PD, reduces PD risk, and generates an oxylipin suggested to hold promise as a PD biomarker. Taken together, current evidence supports aberrant COX‐ and LOX‐derived oxylipin signaling cascades in PD that warrants future research.

The lower amount of aLA C18:3ω3 in PD biological samples, the association between higher aLA C18:3ω3 intake and lower PD risk, and the protective effects of aLA C18:3ω3 supplementation on preventing PD progression in clinical trials suggest that aLA C18:3ω3 metabolism could be a useful target for PD biomarker and therapeutic development. aLA C18:3ω3 effects on PD may be due in part to the biological effects of aLA C18:3ω3‐derived oxylipins and aLA C18:3ω3‐dependent modulation of oxylipin generation from other PUFAs, such as AA C20:4ω6. aLA C18:3ω3, predominantly processed through LOX and CYP pathways, is a potent inhibitor of the pro‐inflammatory COX pathway that appears to be dysregulated in PD [237, 238]. PD‐associated protective effects of aLA C18:3ω3 may be stronger in men with PD, who appear to have greater aLA C18:3ω3‐induced PD risk reduction than women. This may be due in part to lower desaturation and elongation rates of aLA C18:3ω3 in men than women, testosterone‐induced upregulation of COX expression, and a greater anti‐inflammatory response to pharmacological COX inhibition in men than women [177, 178, 179, 221, 222]. As a result, men with PD may benefit more than women with PD from supplementation of COX‐inhibiting aLA C18:3ω3. However, to the best of our knowledge, no pure, human‐grade aLA C18:3ω3 supplements are currently available. Given this limitation, increased consumption of and supplementation with plant sources of ω3 PUFAs, such as flaxseed oil or walnuts that contain high concentrations of aLA C18:3ω3, may be more beneficial for individuals with PD, particularly men with PD, than animal sources of ω3 PUFAs, which are higher in EPA C20:5ω3 and DHA C22:6ω3 that may be less protective in PD than aLA C18:3ω3.

## 4 Future Directions

More research is required to elucidate the role of oxylipin metabolism in PD. Oxylipin research in PD has thus far been limited in part by a relatively small number of laboratories with the technical capabilities to quantify oxylipins that exist endogenously in the nanomolar or picomolar range in human biological samples, and methodological limitations like sample handling and long sample storage durations prior to oxylipin quantification that precipitate ex vivo oxidation and oxylipin artefacts in biological samples. Oxylipin profiling studies are increasing due to heightened interest in oxylipins in the growing neurolipidomics field and advancements in oxylipin quantification protocols and standardized recommendations [239]. Future studies should determine (1) the oxylipin‐ome in PD and (2) how alterations in oxylipin metabolism contribute to PD pathogenesis. Oxylipin metabolism is virtually unexplored in genetic forms of PD. Oxylipin profiling studies of different biological samples (e.g., plasma, CSF, postmortem brain tissue) from males and females with sporadic and genetic forms of PD are needed to understand how the oxylipin‐ome is altered in PD. To date, no study has examined the PD oxylipin profiles in CSF or any brain region beside cerebellar mitochondria. Determining PD‐associated oxylipin changes in the CNS and periphery by coupling brain region‐specific oxylipin‐ome differences in affected and unaffected PD brain regions to PD‐associated disturbances in CSF and plasma oxylipin profiles of the same individuals is critical for the identification of novel PD biomarkers in biofluids that reflect PD pathologies in the brain. This would support the monitoring of PD progression and ultimately the assessment of PD therapeutic efficacy. PD biomarker development will be further supported by PD longitudinal studies quantifying oxylipins from the same individuals over time and by examining the relationship between PD‐associated alterations in oxylipin profiles and markers of PD progression (e.g., UPDRS and H&Y scale scores). As PUFA supplementation alters human oxylipin profiles and because PD clinical trials have found that PUFA supplementation may impact PD progression, future PD clinical trials utilizing PUFA supplementation should consider incorporating oxylipin profiling into the study design in order to identify shifts in the oxylipin‐ome that are associated with alterations to PD clinical outcomes [106, 107, 109]. To the best of our knowledge, no study has reported concentrations of esterified (>90% oxylipins [103]) or total (non‐esterified + esterified) oxylipins in PD or PD samples. Notably, total oxylipin concentrations in clinical samples are more stable and more resistant to artificial oxylipin production and degradation during sample storage than concentrations of non‐esterified oxylipins, the only form of oxylipins that have been profiled in PD studies thus far [109].

As PD oxylipin‐omic profiling studies are in their infancy, the mechanism of how a disrupted PD oxylipin‐ome affects PD pathologies is not well understood. To address this, future studies should examine how distinct oxylipins produced from different precursor PUFAs and enzymatic pathways impact PD‐associated αS dyshomeostasis and aggregation. It is possible that oxylipins may affect αS pathologies directly via αS‐oxylipin binding or indirectly through oxylipin‐induced inflammatory changes that disrupt αS homeostasis. Future work exploring how different oxylipins affect αS aggregation into Lewy body‐like inclusions, αS cellular localization in the cytosol or on membranes, the proportion of αS in physiological tetramers vs. aggregation‐prone monomers, and the relative amount of αS phosphorylated at pS129 will be important. Moreover, because of the emerging bidirectional relationship between oxylipin metabolism and gut microbiota, dysregulated oxylipin signaling cascades may also affect PD pathologies through alterations in the gut‐brain axis [240, 241, 242]. Due to the highly dynamic nature of oxylipin metabolism, time course studies will be crucial for understanding the mechanism of how oxylipins affect PD pathologies. Elucidation of the aberrant PD oxylipin‐ome could identify novel dysregulated FA signaling pathways in PD and support the development of novel PD biomarkers and disease‐modifying therapeutics.

## Conflicts of Interest

SF is an ad hoc consultant to Janssen and to Congruence Therapeutics.

## Supporting information




**Supporting File**: advs75269‐sup‐0001‐SuppMat.docx.
