# Supplementary material for: The Emerging Parkinson's Disease Oxylipin‐Ome
Source: Adv Sci (Weinh). 2026 Apr 22;13(33):e22997. doi: 10.1002/advs.202522997 (PMC13271650; doi:10.1002/advs.202522997)
Supplement: Supplementary file 1 — Supporting File: advs75269‐sup‐0001‐SuppMat.docx. [file ADVS-13-e22997-s001.docx]

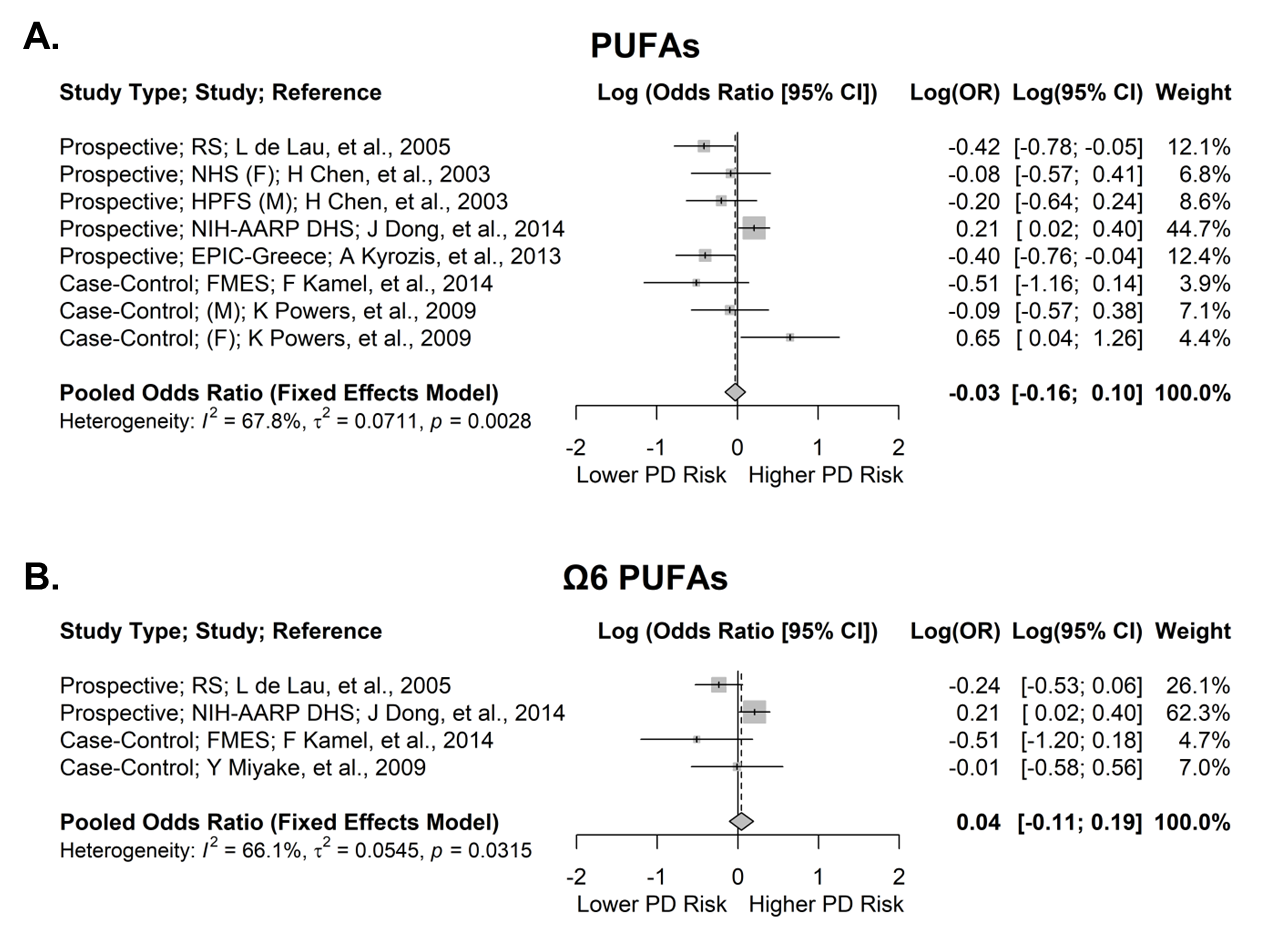
**Supplementary Figure 1. Meta-Analyses on Dietary Consumption of Grouped Polyunsaturated Fatty Acid (PUFA)s and Parkinson’s Disease (PD) Risk.** Forest plots indicate the log odds ratios (OR), 95% confidence intervals (CI), and study weights of reports analyzed in the meta-analysis to examine the impact of dietary PUFA intake on PD risk. Horizontal lines in the plots correspond to the 95% CI and the square on each line represents the weight of that study on the overall pooled OR as calculated using a fixed effects model for assessing how PUFA (**A**) and omega (ω)6 PUFA intake (**B**) affect PD risk.

**
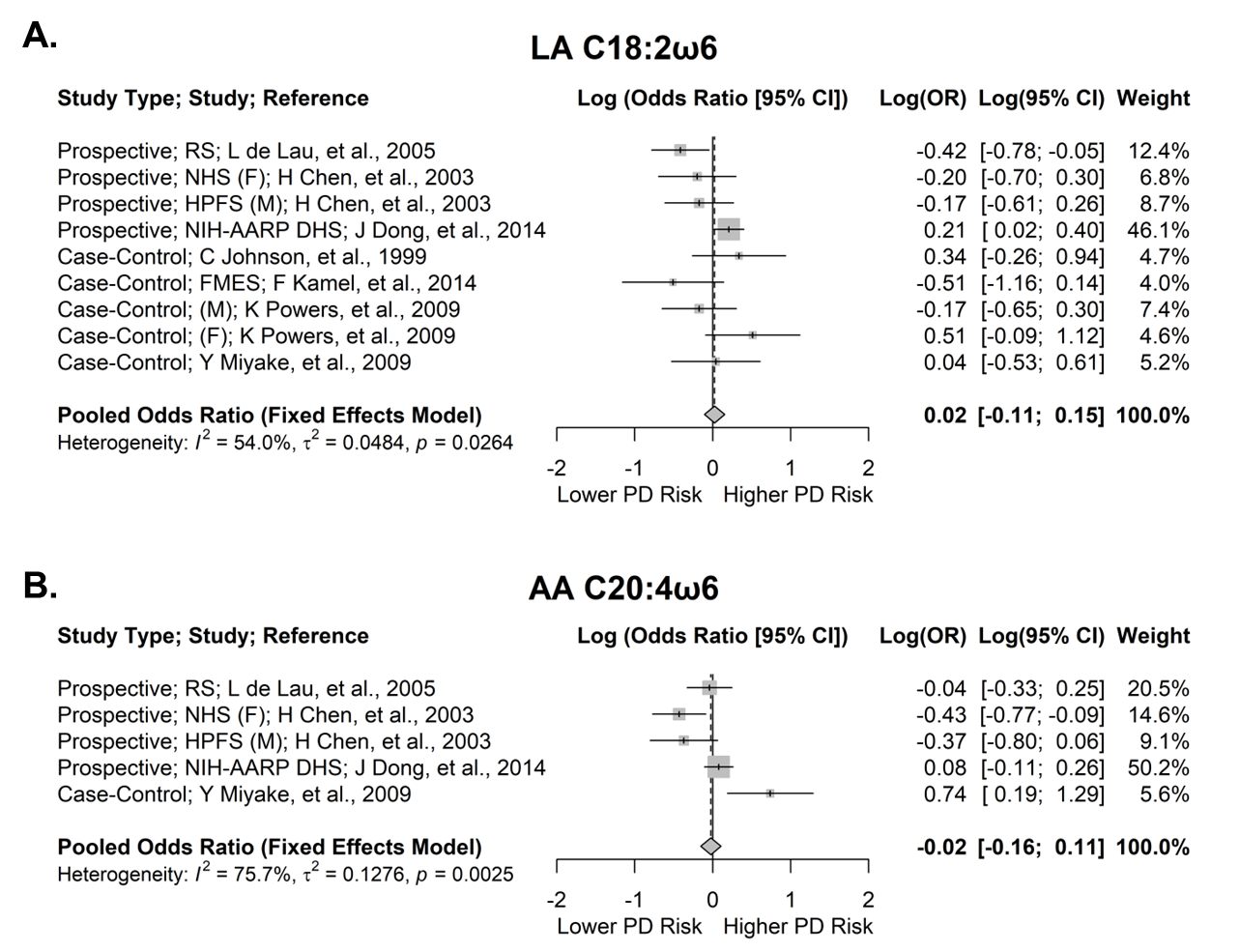
**


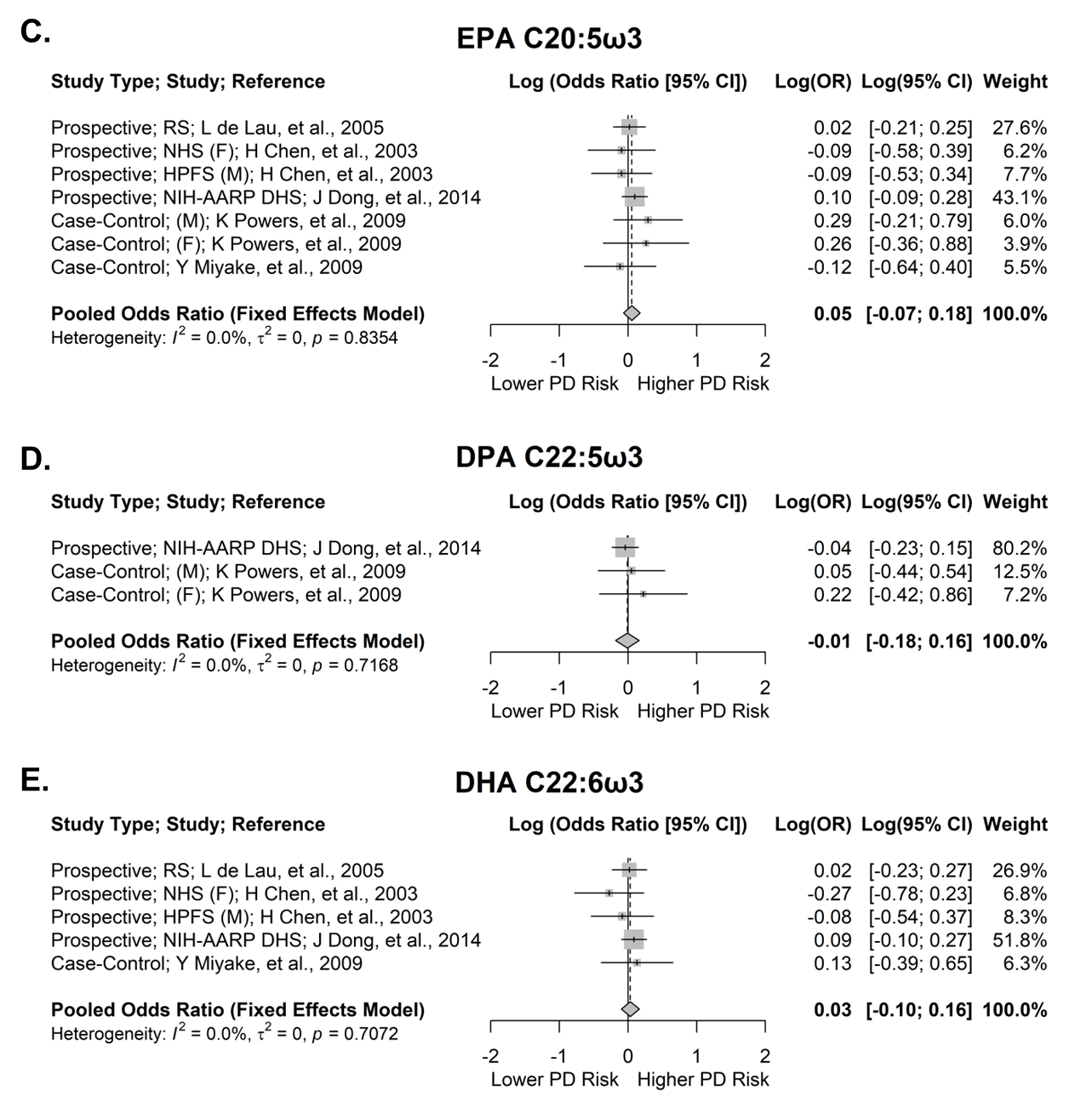
**Supplementary Figure 2. Meta-Analyses on Dietary Consumption of Individual Polyunsaturated Fatty Acid (PUFA)s and Parkinson’s Disease (PD) Risk.** Forest plots indicate the log odds ratios (OR),95% confidence intervals (CI), and study weights of reports analyzed in the meta-analysis to examine the impact of dietary PUFA intake on PD risk. Horizontal lines in the plots correspond to the 95% CI and the square on each line represents the weight of that study on the overall pooled OR as calculated using a fixed effects model for assessing how intake of linoleic acid (LA) C18:2ω6 (**A**), arachidonic acid (AA) C20:4ω6 (**B**), eicosapentaenoic acid (EPA) C20:5ω3 (**C**), docosapentaenoic acid (DPA) C22:5ω3 (**D**), and docosahexaenoic acid (DHA) C22:6ω3 (**E**) affects PD risk.

**
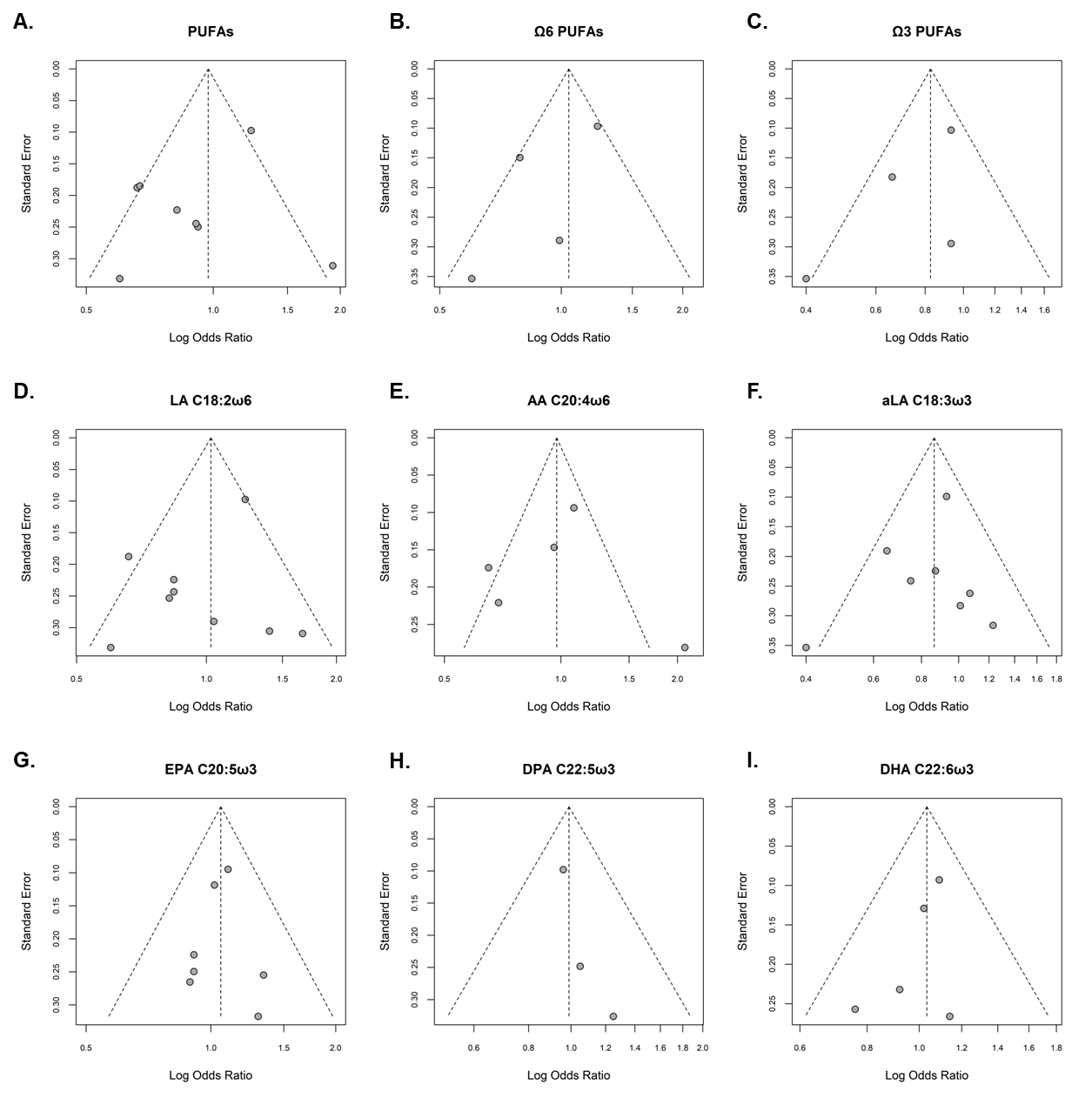
Supplementary Figure 3. Meta-Analysis Funnel Plots of Polyunsaturated Fatty Acid (PUFA) Dietary Intake and Parkinson’s Disease (PD) Risk.** Funnel plots demonstrate the log odds ratios and standard error reported in each studies included in meta-analyses assessing the impact of dietary PUFA intake on PD risk. LA: linoleic acid C18:2ω6. AA: arachidonic acid C20:4ω6. EPA: eicosapentaenoic acid C20:5ω3. DPA: docosapentaenoic acid C22:5ω3. DHA: docosahexaenoic acid C22:6ω3.

**
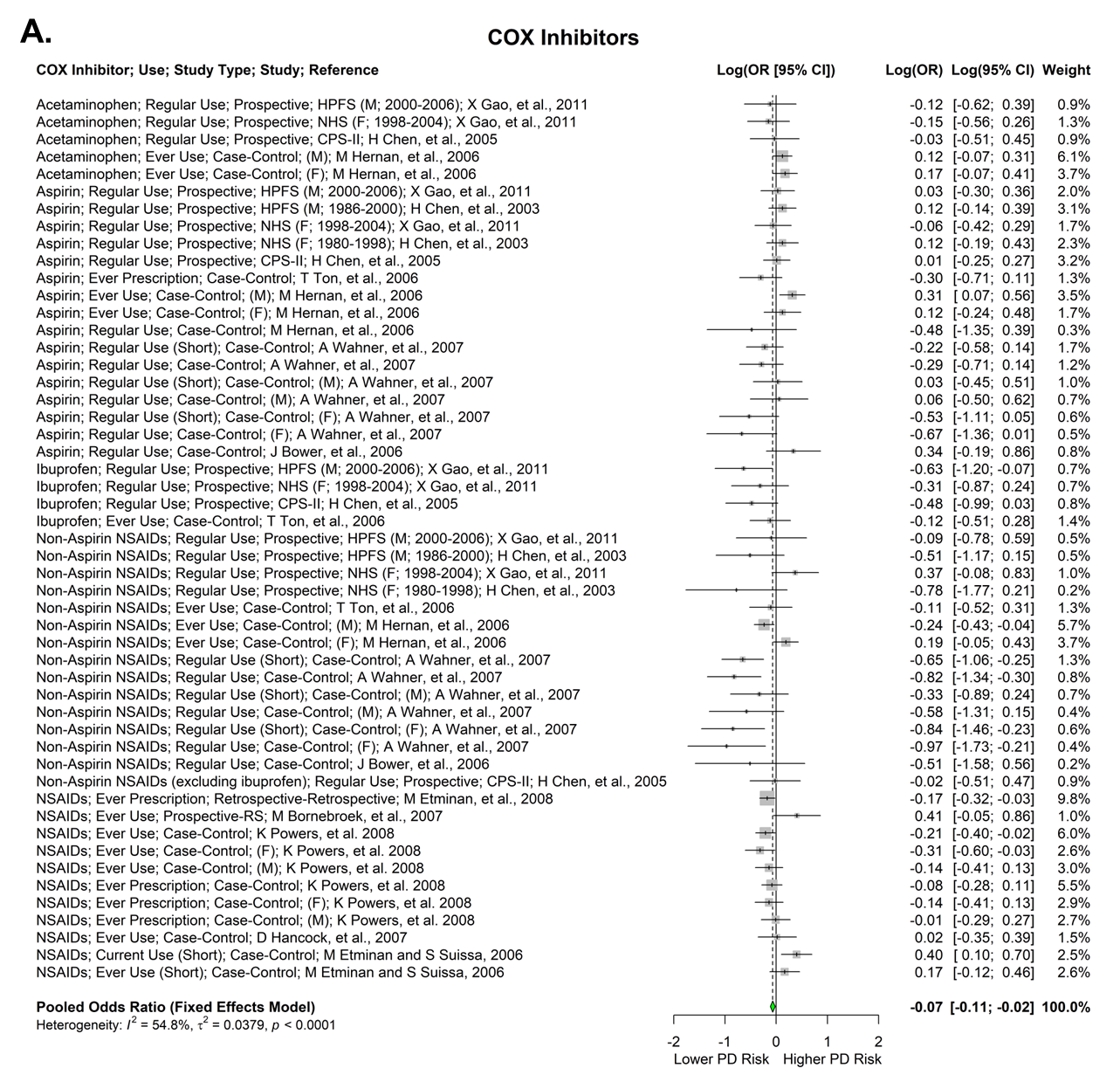
**

**
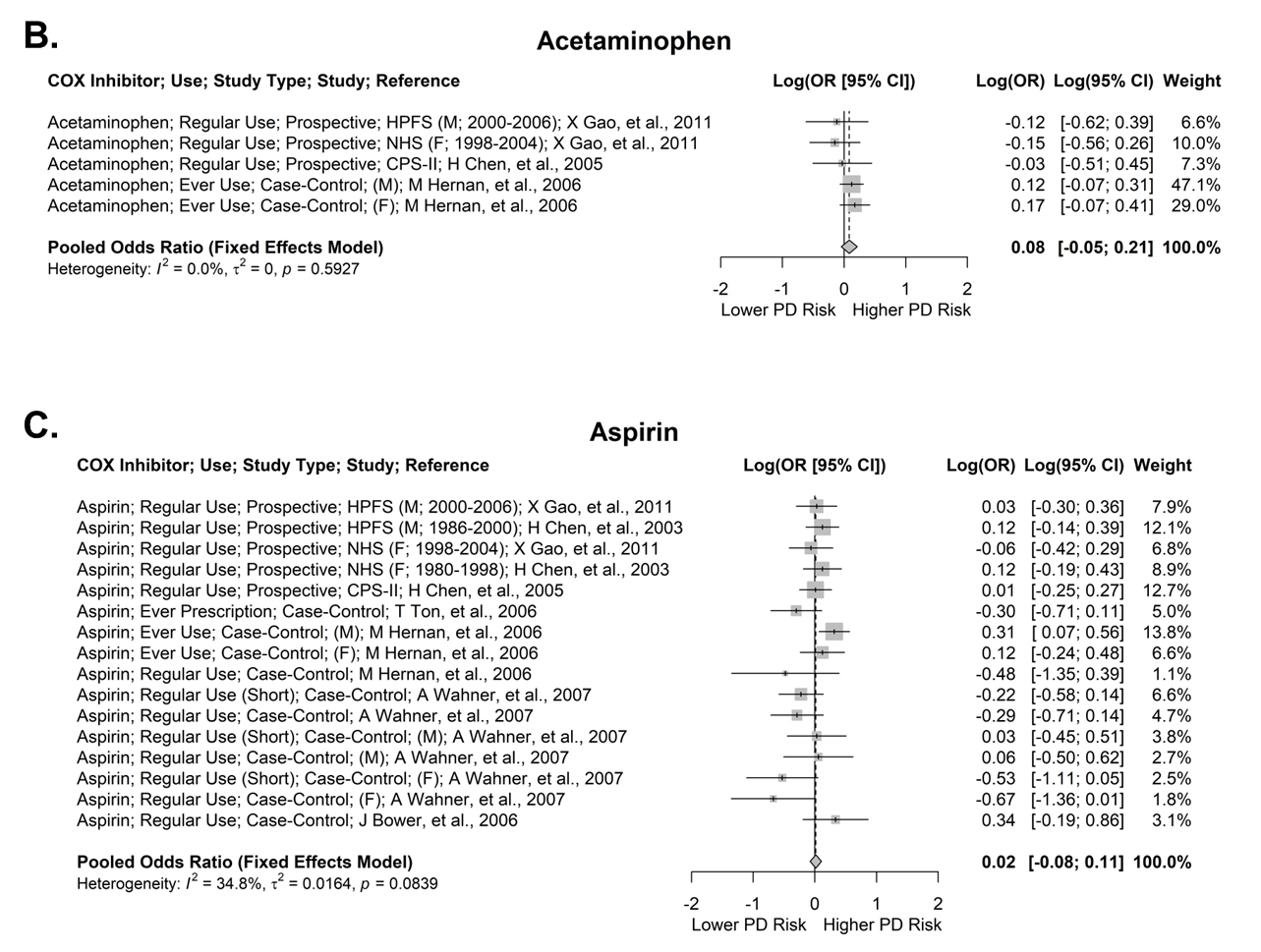
**

**Supplementary Figure 4. Cyclooxygenase (COX) Inhibitor Use May Reduce Parkinson’s Disease Risk.** Forest plots indicate the log odds ratios (OR),95% confidence intervals (CI), and study weights of reports analyzed in the meta-analysis to examine how PD risk is impacted by all types of COX inhibitor use(**A**), acetaminophen use (**B**), or aspirin use (**C**). Horizontal lines in the plots correspond to the 95% CI and the square on each line represents the weight of that study on the overall pooled OR. The green diamond at the bottom in (**A**) shows the significantly lower pooled OR with 95% CI as calculated using a fixed effects model for assessing how COX inhibitor use affects PD risk. NSAID: non-aspirin non-steroidal anti-inflammatory drug.

**
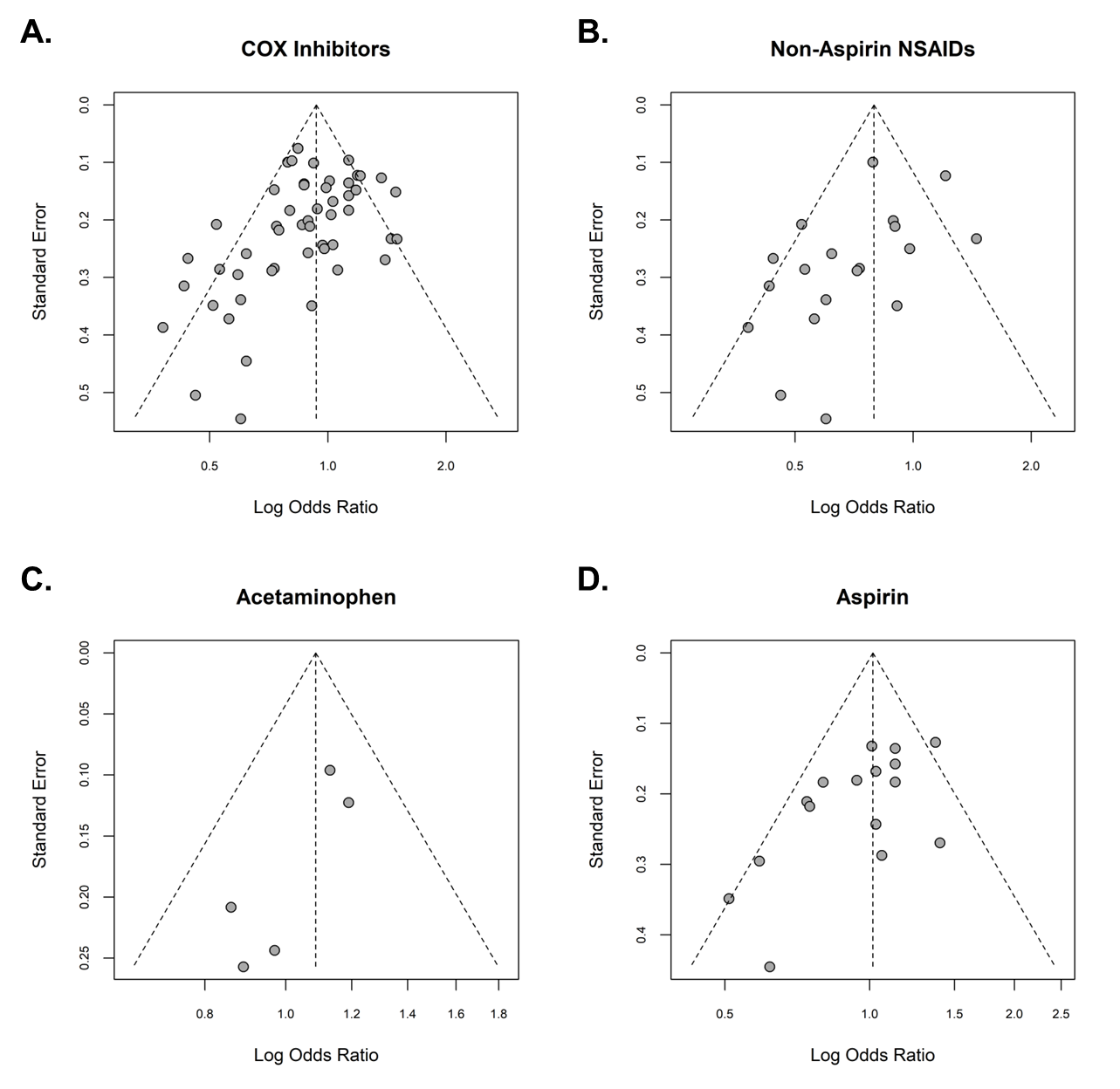
Supplementary Figure 5. Meta-Analysis Funnel Plots of Cyclooxygenase (COX) Inhibitor Use and Parkinson’s Disease (PD) Risk.** Funnel plots demonstrate the log odds ratios and standard error reported in each studies included in meta-analyses assessing the impact of COX inhibitor use on PD risk. NSAID: non-aspirin non-steroidal anti-inflammatory drug.
